# The specificity of HEG1 as mesothelioma marker depends on the differential diagnosis

**DOI:** 10.1007/s00428-026-04438-w

**Published:** 2026-02-09

**Authors:** Ben Davidson, Lara Maria Stričak, Arild Holth, Annette Torgunrud, Jeremias Wohlschlaeger, Martin Tötsch, Assia Bassarova

**Affiliations:** 1 Department of Pathology, Oslo University Hospital, Norwegian Radium Hospital, N-0310 Oslo, Norway; 2 Faculty of Medicine, Institute of Clinical Medicine, University of Oslo, N-0316 Montebello, Oslo Norway; 3 Department of Tumor Biology, Oslo University Hospital, Oslo, Norway; 4 Institute of Pathology, Academic Teaching Hospitals Flensburg, DIAKO Hospital gGmbH, Flensburg, Germany; 5 Department of Pathology, University Hospital Essen, University of Duisburg-Essen, Essen, Germany; 6 Institute of Cytology, KAGes, 8036 Graz, Austria; 7 University of Zagreb, Zagreb, Croatia

**Keywords:** HEG1, Immunohistochemistry, Diagnosis, Mesothelioma, Tubo-ovarian carcinoma

## Abstract

The objective of this study was to analyze the diagnostic role of HEG homolog 1 (HEG1) in cancers affecting the serosal cavities. HEG1 protein expression by immunohistochemistry was analyzed in 534 specimens (341 effusions and 193 surgical specimens). Effusions consisted of 151 tubo-ovarian carcinomas, 59 breast carcinomas, 44 mesotheliomas, 37 lung carcinomas, 29 uterine corpus and cervical carcinomas, 17 gastrointestinal carcinomas and 4 genitourinary carcinomas. Surgical specimens consisted of 139 tubo-ovarian carcinomas, 42 mesotheliomas, 7 multicystic mesothelial proliferations and 5 papillary mesothelial tumors. HEG1 expression was found in 43/44 (98%) mesothelioma effusions and 39/42 (93%) surgical mesothelioma specimens, as well as all multicystic and papillary mesothelial tumors. HEG1 was infrequently expressed in breast carcinoma (4/59; 7%), lung carcinoma (2/37; 5%) and cervical/uterine carcinoma effusions (3/29; 10%), but was often detected in tubo-ovarian carcinoma effusions (80/151; 53%) and surgical specimens (99/139, 71%). HEG1 was additionally consistently expressed by reactive mesothelial cells in effusions and in endothelial cells in surgical specimens. HEG1 had sensitivity of 95% for diagnosing malignant mesothelioma in all studied specimens, with a specificity of 38% in the differential diagnosis from tubo-ovarian carcinoma and 93% in the differential diagnosis from non-tubo-ovarian carcinomas. Of 179 HEG1-positive carcinomas, 172 expressed the epithelial marker claudin-4. In conclusion, HEG1 is a highly sensitive marker of both benign and malignant mesothelial cells. It shows high specificity in the differentiation of mesothelioma from lung or breast carcinoma but is of little value in differentiating mesothelioma from tubo-ovarian carcinoma. The potential role of HEG1 as vascular marker merits further research.

## Introduction

Mesothelioma is a rare cancer with origin in the serosal cavities. It occurs more commonly in the pleural cavity, but also affects the peritoneum and, more rarely, the pericardium. In 2022, 30,618 new cases and 25,372 deaths from this cancer were reported globally, of whom approximately two-thirds were males [1].

The differentiation of mesothelioma from reactive mesothelial cells and from carcinomas that metastasize to the serosal cavities has for many years been challenging. However, in recent years the use of new markers has resulted in better accuracy in both differential diagnoses [2].

Tsuji et al. identified sialylated protein HEG homolog 1 (HEG1), a mucin-like membrane protein, as a novel, highly specific mesothelioma marker. Using the SKM9-2 monoclonal antibody, membrane, and occasionally cytoplasmic staining was observed in all morphological variants of this tumor, i.e. epithelioid, biphasic, sarcomatoid and desmoplastic mesothelioma. It was absent in non-malignant mesothelial cells. Staining was observed in only 3/310 non-mesothelioma malignancies of various type [3].

Subsequent studies have generally confirmed these findings, though with a lesser degree of specificity with respect to non-pulmonary carcinomas [4–7]. However, the number of tumors of breast or female genital origin included in these studies was relatively small.

Given the fact that both low-grade and high-grade tubo-ovarian serous carcinomas (LGSC; HGSC) express many of the proteins which mesotheliomas stain positive for, including WT1, D2-40, mesothelin, CA 125 and other markers [8], we wished to study whether HEG1 can be used as a specific mesothelial marker in the differential diagnosis from carcinomas affecting the serosal cavies, particularly tubo-ovarian serous carcinomas.

## Material and methods

### Patients and specimens

The series studied consisted of 341 malignant effusions. The majority of effusions (*n* = 299) were submitted to the Department of Pathology at the Norwegian Radium Hospital, Oslo University Hospital, for routine diagnostic purposes during the period 1998–2020. The remaining specimens were from the Departments of Pathology in Graz, Austria (*n* = 22) and Essen, Germany (*n* = 20).

Effusions were tapped from the peritoneal (*n* = 193), pleural (*n* = 140) and pericardial (1) cavities; 7 specimens from Graz with unknown anatomic location. Specimens included 151 tubo-ovarian carcinomas, 59 breast carcinomas, 44 mesotheliomas, 37 lung carcinomas, 29 uterine corpus and cervical carcinomas, 17 gastrointestinal carcinomas and 4 genitourinary carcinomas (Table 1).

**Table 1:** Tumor type and anatomic localization

Tumor type	Anatomic compartment				Total
	Peritoneal	Pleural	Pericardial	Unknown ^*a*^	
Tubo-ovarian carcinoma	146	4	0	1	151
Breast carcinoma	6	50	1	2	59
Mesothelioma	11	33	0	0	44
Lung carcinoma	0	36	0	1	37
Uterine corpus carcinoma	18	3	0	0	21
Uterine cervical carcinoma	6	2	0	0	8
Esophageal carcinoma	0	6	0	0	6
Gastric carcinoma	4	0	0	1	5
Colon carcinoma	1	2	0	1	4
Urothelial carcinoma	1	2	0	0	3
Pancreatic carcinoma	0	1	0	1	2
Prostate carcinoma	0	1	0	0	1
Total	**193**	**140**	**1**	**7**	**341**

^*a*^Seven specimens from Graz with no detail regarding anatomic location

Tubo-ovarian carcinomas consisted of 58 HGSC, 45 LGSC, 20 clear cell carcinomas (CCC), 18 carcinosarcomas (CS), 5 endometrioid carcinoma (EC), 3 carcinomas of mixed type and 2 mucinous carcinomas (MC).

Mesothelioma specimens were all of epithelioid or biphasic type. The majority of lung carcinomas (*n* = 34) were adenocarcinomas, the remaining cases consisting of 2 small cell carcinomas and 1 squamous cell carcinoma. Metastases of uterine corpus origin were from high-grade tumors (serous carcinoma, clear cell carcinoma or the epithelial component of carcinosarcoma). Uterine cervical tumors were adenocarcinomas, though HPV status was not available.

Effusions were centrifuged and cell blocks were made using the thrombin clot method. Diagnoses were established by an experienced cytopathologist (BD) based on morphological evaluation of smears and hematoxylin and eosin-stained sections from cell blocks, in combination with IHC applying established antibodies against epithelial and mesothelial epitopes [8].

Following staining results in the effusion series, the study was expanded to include surgical specimens consisting of 139 tubo-ovarian carcinomas and 42 mesotheliomas from patients operated at the Norwegian Radium Hospital. The former consisted of ovarian, omental or peritoneal specimens from patients diagnosed with HGSC (*n* = 113), LGSC (*n* = 12), CCC (*n* = 8), EC (*n* = 4) and CS (*n* = 2). Mesotheliomas consisted of 25 pleural and 17 peritoneal tumors, of which 34 were of epithelioid type and 8 were biphasic. In addition, 7 multicystic mesothelial proliferations and 5 papillary mesothelial tumors were studied. Tumors were diagnosed by an experienced surgical pathologist (BD) based on current guidelines.

Informed consent was obtained according to national and institutional guidelines. Study approval was given by the Regional Committee for Medical Research Ethics in Norway (S-04300). The multicystic and papillary mesothelial tumors are part of a peritoneal malignancy biobank with separate ethics authorization (S-07160b). Ethics approval for specimens from Essen and Graz was obtained according to institutional guidelines at these hospitals.

### Immunohistochemistry

Formalin-fixed, paraffin-embedded sections from the above-described 341 effusions and 181 surgical specimens were analyzed for HEG1 protein expression using the Dako EnVision Flex + System (K8012; Dako/Agilent, Glostrup, Denmark). The HEG1 antibody was a mouse monoclonal antibody (clone SKM9-2) purchased from MyBioSource (San Diego CA, USA). A 1:100 dilution was used, with antigen retrieval in pH 6 buffer.

The majority (179 of 189) of HEG1-positive carcinomas were additionally stained for claudin-4 using a mouse monoclonal antibody (clone 3E2C1) purchased from Zymed Laboratories Inc. (San Francisco CA, USA). A 1:600 dilution was used, with antigen retrieval in pH 9 buffer.

Following deparaffinization and antigen retrieval, sections were incubated with the primary antibody (30 min), treated with EnVision™ Flex + mouse linker (15 min) and EnVision™ Flex/HRP enzyme (30 min) and stained for 10 min with 3′3-diaminobenzidine tetrahydrochloride (DAB), counterstained with hematoxylin, dehydrated and mounted in Toluene-Free Mounting Medium (Dako). Positive controls consisted of a surgical specimen of colon adenocarcinoma for HEG1, normal tonsil for claudin-4. Negative controls consisted of slides stained with murine IgG at the same concentration.

IHC scoring: Staining was scored by the corresponding author (BD) using a semi-quantitative 0–4 scale as follows: 0 = no staining, 1 = 1–5%, 2 = 6–25%, 3 = 26–75%, 4 = 76–100% of tumor cells. Only distinct membrane expression was scored as positive. Effusion specimens were additionally scored for HEG1 expression by a second author (LS).

### Statistical analysis

Statistical analysis was performed applying the SPSS-PC package (Version 30). Probability of < 0.05 was considered statistically significant.

The Kruskal–Wallis H test was applied to comparative analyses of HEG1 protein expression by IHC and primary tumor origin, in which the latter were grouped as breast, lung, female genital or gastrointestinal carcinoma or mesothelioma. The same test was applied to comparative analyses of HEG1 expression in different tubo-ovarian carcinoma histotypes. The Mann–Whitney U test was applied to 2-tier analyses comparing expression in mesothelioma vs. female genital carcinomas and to analyses of the association between HEG1 expression and anatomic site in mesothelioma.

## Results

**Fig. 1:**
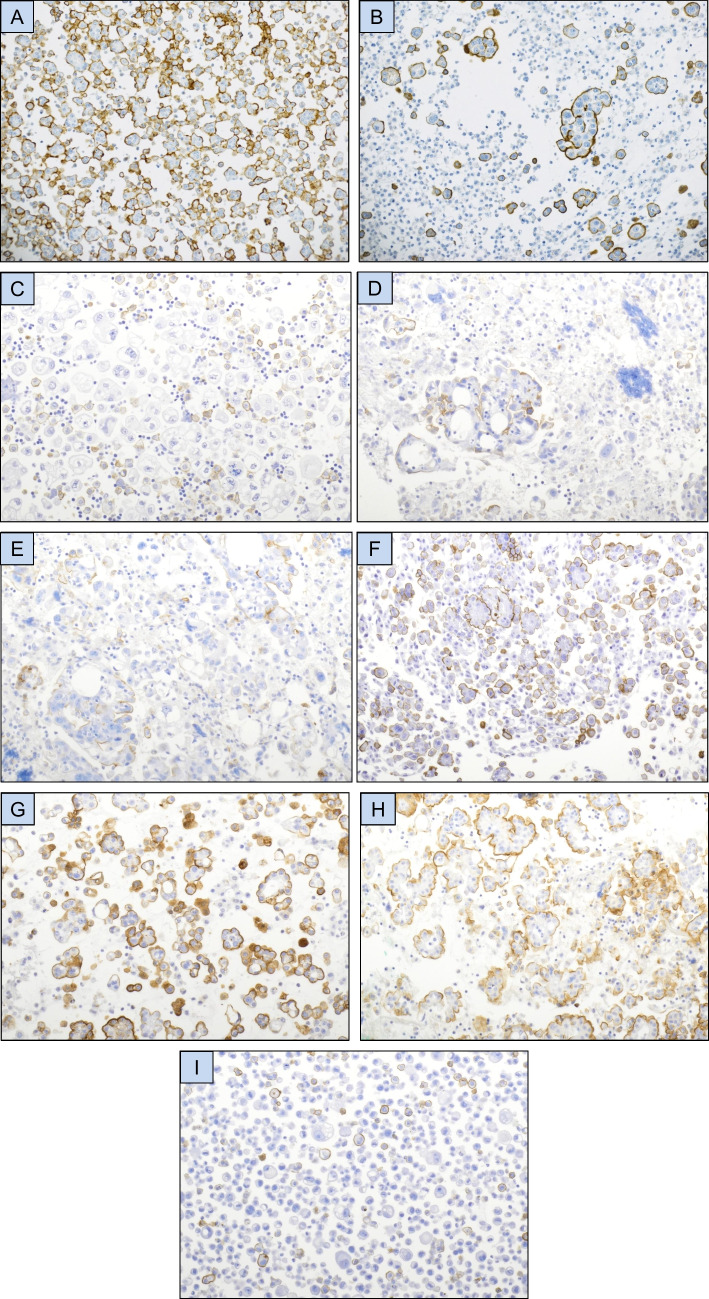

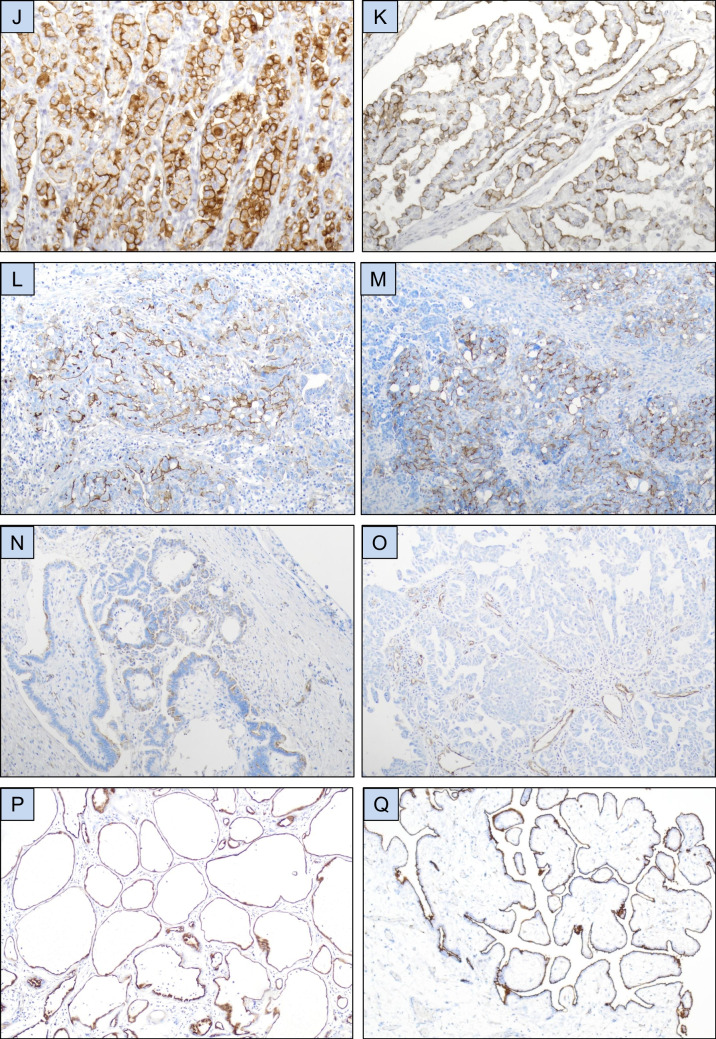
HEG1 immunohistochemistry. (**A**-**B**) HEG1 protein expression in mesothelioma cells in effusion. (**C**) HEG1 expression in reactive mesothelial cells in effusion. (**D**-**H**) HEG1 expression in tubo-ovarian carcinoma effusions: Partial positivity in high-grade serous carcinoma (**D**-**E**), diffuse positivity in low-grade serous carcinoma (**F**), clear cell carcinoma (**G**) and carcinosarcoma (**H**). (**I**) Partial HEG1 expression in colon carcinoma cells in effusion. (**J**-**K**) HEG1 protein expression in surgical specimens of epithelioid mesothelioma. (**L**-**N**) HEG1 protein expression in high-grade (**L**-**M**) and low-grade (**N**) tubo-ovarian serous carcinoma (**O**) HEG1 expression in vessels in high-grade tubo-ovarian serous carcinoma. Tumor cells are negative in this case. (**P**-**Q**) HEG1 expression in a multicystic mesothelial proliferation (**P**) and papillary mesothelial tumor (**Q**)

**Fig. 2:**
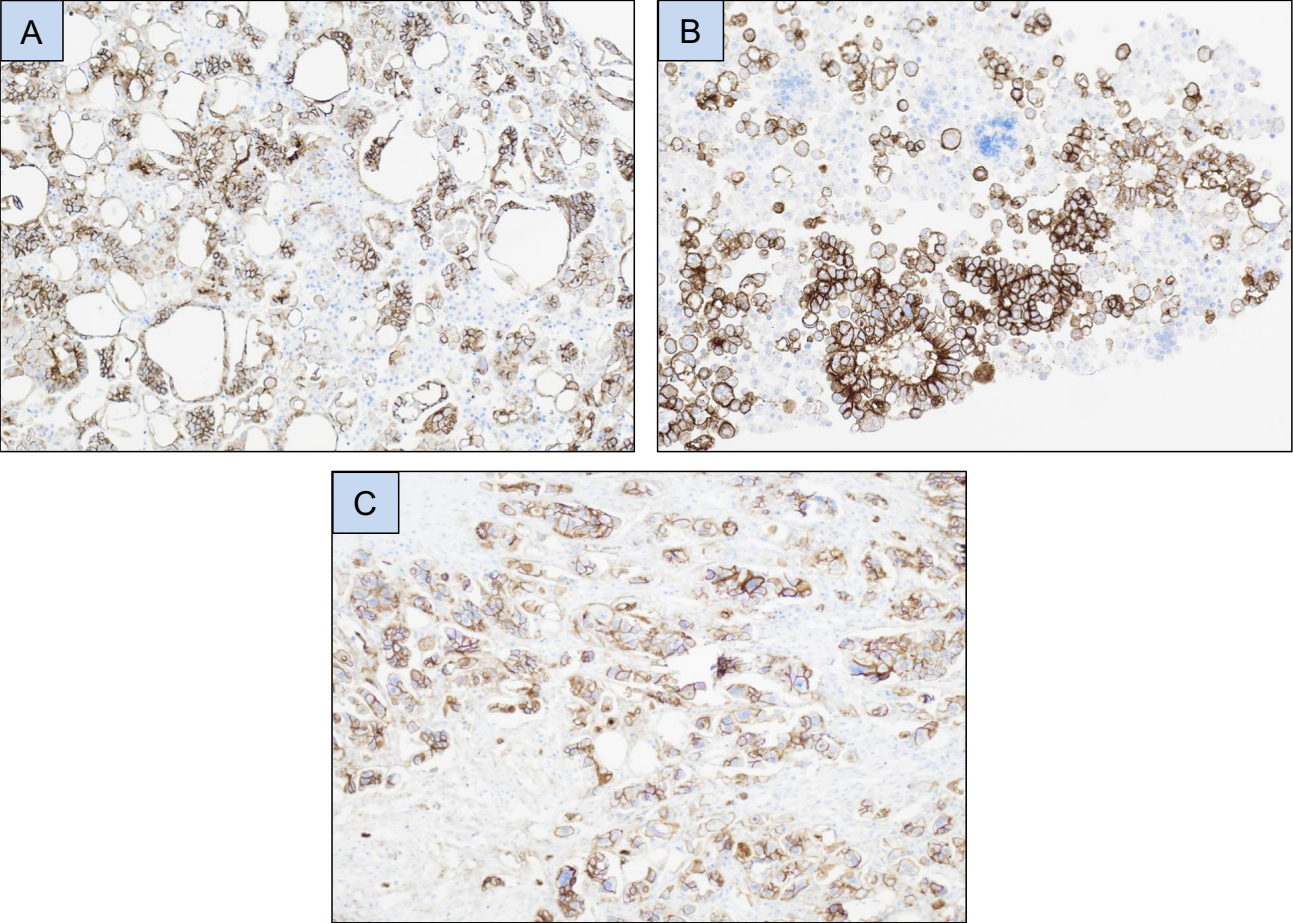
Claudin-4 immunohistochemistry. (**A**-**C**) Claudin-4 expression in a breast carcinoma effusion (**A**), high-grade serous carcinoma (HGSC) effusion (**B**), and HGSC surgical specimen (**C**)

HEG1 was expressed in tumor cells in the majority of mesothelioma effusions, most frequently in > 75% of tumor cells (Figs. 1**-A, 1-B; **Table 2). Staining extent was comparable in pleural and peritoneal mesotheliomas (*p* = 0.334; data not shown). Consistent membrane positivity was seen in reactive mesothelial cells in effusions from patients with metastatic carcinoma, irrespective of expression levels in carcinoma cells (Fig. 1**-C**). Expression in carcinoma cells was frequently seen in tubo-ovarian carcinomas, including all histotypes which metastasize frequently to the serosal cavities, i.e. HGSC, LGSC, CCC and CS (Figs. 1**-D to 1-H; **Tables 2, 3), and was infrequent in tumors of other origin (Fig. 1-I; Table 2 and Fig. 2).

**Table 2:** Staining results in 341 effusions

Tumor type	Staining extent					Total positive (%)	CLD4 +/HEG1 + carcinomas	Total
	0%	1–5%	6–25%	26–75%	76–100%			
Tubo-ovarian carcinoma	71	46	16	15	3	80 (53%)	78/80	151
Breast carcinoma	55	1	2	1	0	4 (7%)	4/4	59
Mesothelioma	1	0	2	7	34	43 (98%)	-	44
Lung carcinoma	35	1	0	1	0	2 (5%)	1/2	37
Uterine corpus carcinoma	18	1	1	0	1	3 (14%)	2/3	21
Uterine cervical carcinoma	8	0	0	0	0	0 (0%)	-	8
Esophageal carcinoma	5	0	0	0	1	1 (17%)	1/1	6
Gastric carcinoma	5	0	0	0	0	0 (0%)	-	5
Colon carcinoma	4	0	0	0	0	0 (0%)	-	4
Urothelial carcinoma	3	0	0	0	0	0 (0%)	-	3
Pancreatic carcinoma	2	0	0	0	0	0 (0%)	-	2
Prostate carcinoma	1	0	0	0	0	0 (0%)	-	1

**Table 3:** HEG1 taining results in 151 tubo-ovarian carcinoma effusions based on histotype

Tumor type	Staining extent					Total positive (%)	Total
	0%	1–5%	6–25%	26–75%	76–100%		
High-grade serous carcinoma	25	15	10	7	1	33 (57%)	58
Low-grade serous carcinoma	16	19	5	5	0	29 (64%)	45
Clear cell carcinoma	13	5	0	1	1	7 (35%)	20
Carcinosarcoma	9	5	1	2	1	9 (50%)	18
Endometrioid carcinoma	3	2	0	0	0	2 (40%)	5
Mucinous carcinoma	2	0	0	0	0	0 (0%)	2
Carcinoma of mixed histotype	3	0	0	0	0	0 (0%)	3

Comparative analysis of HEG1 expression mesothelioma vs. metastatic carcinoma showed significant overexpression in the former (*p* < 0.001). Comparative analysis of HEG1 expression in the 4 above-mentioned tubo-ovarian carcinoma histotypes did not show significant differences (*p* = 0.252).

In view of HEG1 staining results in effusions, expression of this marker was analyzed in surgical specimens from patients diagnosed with tubo-ovarian carcinoma (*n* = 139), the majority of which consisted of HGSC, and mesothelioma (*n* = 42). As in effusions, near-universal expression of HEG1 was seen in mesotheliomas cells (Figs. 1**-J, 1-K**), but also in a significant percentage of tubo-ovarian carcinomas (Figs. 1**-L to 1-N; **Table 4). Consistent expression of this protein was seen in endothelial cells, irrespective of staining in tumor cells (Fig. 1**-O**).

**Table 4:** Staining results in mesothelioma (*n* = 42) and tubo-ovarian carcinoma (*n* = 139) surgical specimens

Tumor type	Staining extent					Total positive (%)	CLD4 +/HEG1 + carcinomas	Total
	0%	1–5%	6–25%	26–75%	76–100%			
Mesothelioma	3	4	5	9	21	39 (93%)	-	42
HGSC	29	53	22	9	0	84 (74%)	75/77^*a*^	113
LGSC	2	2	5	3	0	10 (83%)	9/9^*b*^	12
CCC	5	3	0	0	0	3 (37%)	2/3	8
EC	3	0	1	0	0	1 (25%)	NA^*c*^	4
CS	1	0	1	0	0	1 (50%)	1/1	2

*CLD4*  claudin-4, *HGSC* high-grade serous carcinoma, *LGSC* low-grade serous carcinoma, *CCC* clear cell carcinoma, *EC* endometrioid carcinoma, *CS* carcinosarcoma, *NA* not avaiable

^*a*^Material unavailable for CLD4 staining in 7 cases

^*b*^Material unavailable for CLD4 staining in 1 case

^*c*^Material unavailable for CLD4 staining

As in mesothelioma effusions, no significant difference in HEG1 expression was observed between pleural and peritoneal mesotheliomas (p = 0.549; data not shown).

All multicystic mesothelial proliferations and papillary mesothelial tumors were HEG1-positive (Fig. 1**-P, 1-Q**).

Though based on a small number of LGSC and CCC, comparative analysis of HEG1 expression in different tubo-ovarian carcinoma histotypes showed significantly higher expression in HGSC and LGSC compared to CCC (*p* = 0.004).

HEG1 had sensitivity of 95% for diagnosing mesothelioma in all studied specimens, with a specificity of 38% in the differential diagnosis from tubo-ovarian carcinoma and 93% in the differential diagnosis from non-tubo-ovarian carcinomas.

Given the suggested role for claudin-4 as a carcinoma marker, to be used in conjunction with HEG1 in the differential diagnosis between mesothelioma and carcinoma [7], we stained all HEG1-positive carcinomas for which additional slides were available for claudin-4. These consisted of all 90 effusions with carcinomas of various origin that had any extent of HEG1 expression, and 89/99 surgical specimens of tubo-ovarian carcinoma.

In effusions, results by score were as follows: 0: 4 cases; 1: 2 cases; 2: 9 cases; 3: 24 cases; 4: 51 cases. In surgical specimens, staining was as follows: 0: 3 cases; 1: 10 cases; 2: 13 cases; 3: 34 cases; 4: 29 cases. Overall, 172/179 carcinomas (96%) expressed claudin-4.

## Discussion

Epithelial markers that are expressed in the majority of metastatic carcinomas, such as claudin-4 and Ber-EP4, effectively differentiate these tumors from mesothelioma and reactive mesothelial cells, though Ber-EP4 is focally expressed in a substantial number of epithelioid mesotheliomas. Several robust mesothelioma markers, including positive staining for calretinin, combined with loss of BAP1 and MTAP, currently make the diagnosis of this cancer more certain [2, 8].

Tubo-ovarian or uterine serous carcinomas rarely express calretinin more than focally and do not show loss of BAP1 or MTAP. They additionally commonly express PAX8 and TAG72/B72.3, both of which are infrequently (PAX8) or rarely (TAG-72) expressed in mesothelioma. Nevertheless, serous carcinomas and mesotheliomas do share the expression of a large number of markers used in the diagnostic work-up of serosal cancers. Marker performance consequently depends on the differential diagnosis, as well as anatomic location. For example, the breast carcinoma marker trichorhinophalangeal syndrome type 1 (TRPS1) is highly specific in the differential diagnosis from lung carcinoma or mesothelioma, but not from tubo-ovarian carcinoma [9]. MTAP is often lost in pleural mesothelioma, but less frequently in its peritoneal counterpart [10].

In the present study, we analyzed the diagnostic role of HEG1 as mesothelioma marker, with focus on the differential diagnosis from metastatic carcinoma. The SKM9-2 monoclonal antibody, first isolated by Tsuji et al. and used in subsequent studies, and now commercially available, was used in the present study.

In the initial report by Tsuji et al. [3], analysis was performed on tissue microarrays (TMA). Use of HEG1 showed 99% specificity and 92% sensitivity in diagnosing mesothelioma. RMC were negative. Non-mesothelioma malignancies were almost universally negative. Of note, the 10 tumors designated as ‘ovary adenocarcinoma’ without further detail were uniformly negative for HEG1, but given the fact that only 3 were WT1-positive, consisted probably partly of non-serous carcinomas.

The TMA-based study by Naso et al. [4] similarly included a large number of lung carcinomas of different histotype, all of which were negative for HEG1, whereas 3/17 HGSC were positive.

Another study by Hiroshima et al. [5] analyzed serous effusions. HEG1 stained 100% of mesotheliomas and 76.9% of RMC specimens, compared to 6/21 ovarian carcinomas.

A large subsequent study of surgical specimens by the same author [6], using both whole sections and TMAs, again highlighted the frequent expression of HEG1 in mesotheliomas of all histological variants, though staining was most frequent in epithelioid tumors. Lung and breast adenocarcinomas were negative, but cytoplasmic HEG1 expression was seen in some squamous cell lung carcinomas. Staining was found in 6/9 tumors designated as ovarian serous carcinoma and 2/6 uterine cervical carcinomas.

A more recent study, written as review, combined results from the above studies with some new cases and focused on mesotheliomas (*n* = 434) and non-small cell lung carcinomas (*n* = 360). HEG1 had sensitivity and specificity of 91% and 99.7%, respectively, and its use combined with claudin-4, which had sensitivity of 93% and specificity of 98.9%, was sufficient to classify the majority of cases [7].

Results from references 3–6, with focus on the major diagnostic categories in the present study, are summarized in Table 5.

**Table 5:** HEG1 staining results in previous studies

Ref # 3	**Mesothelioma**	**Lung carcinoma**	**Breast carcinoma**	**Ovarian carcinoma**	**Other carcinomas + carcinosarcoma**	**Other cancers**
	119/130 (92%)	0/98 (0%)	0/10 (0%)	0/10 (0%)	1/50 (2%)	2/142 (1%)
Ref # 4	**Mesothelioma**	**Lung carcinoma**	**Breast carcinoma**	**HGSC**	**Other cancers**	**Reactive mesothelium**
	79/101 (78%)	0/167 (0%)	-	3/17 (18%)	0/10 (0%)	35/40 (88%)^*a*^
Ref # 5	**Mesothelioma**	**Lung carcinoma**	**Ovarian carcinoma**^*b*^	**Other carcinomas**	**Other cancers**	**Reactive mesothelium**
	41/41 (100%)	0/26 (0%)	6/21 (29%)	0/7 (0%)	0/1 (0%)	20/26 (77%)
Ref # 6	**Mesothelioma**^*c*^	**Lung carcinoma**	**Breast carcinoma**	**Ovarian carcinoma**	**Other carcinomas**	**Other cancers**
	112/122 (92%)	11/75 (15%)	0/7 (0%)	6/9 (67%)	6/39 (15%)	13/16 (81%)

^*a*^Epithelioid morphology

^*b*^Including 11 serous carcinomas, 4 CCC and 6 ‘NOS’. Staining was seen in 6 serous carcinomas and none of the other groups

^*c*^Whole sections

Staining conditions in references # 3–6 and the current study are detailed in Table 6. Reference # 7 is a review that appears to summarize previous data, to which new cases were added, and has no Materials and Methods section.

**Table 6:** HEG1 staining conditions in previous studies

Ref #	Staining platform	Concentration/Dilution	Incubation time	Antigen retrieval	Detection system
3	Histostainer 48 A (Nichirei Co., Tokyo, Japan	NS^*a*^	NS	121 °C for 10 min in Citrate pH 6	EnVision + (Dako)
4	Omnis autostainer (Dako)	1:200	20 min	pH 6.1 retrieval buffer for 40 min	EnVision FLEX + (Dako)
5	Leica BOND Max automatic stainer and Leica BOND III automatic stainer (Leica Biosystems)	20 μg/mL	NS	121 °C for 10 min in Citrate pH 6	NS
6	Leica BOND Max automatic stainer (Leica Biosystems)	20 μg/mL	NS	121 °C for 10 min in Citrate pH 6	NS
Current study	Manual	1:100	30 min	Citrate pH 6	EnVision FLEX + (Dako)

^*a*^NS = not specified

Given the above suggestion to combine HEG1 and claudin-4 in the differential diagnosis of mesothelioma from carcinoma, we stained the majority of HEG1-positive carcinomas for claudin-4. Of 179 tumors stained, 172 (96%) expressed claudin-4, suggesting that the latter is indeed a robust carcinoma marker even in HEG1-positive tumors. Though not statistically analyzed, the percentage of tumors showing claudin-4 expression in > 25% of tumor cells was higher in tubo-ovarian carcinoma effusions compared to surgical specimens (68/80, 85% vs. 63/89, 71%), in agreement with a previous study by our group [11].

The present study is in agreement with the above-discussed previous studies with respect to the sensitivity of HEG1 in diagnosing mesothelioma and its specificity in the differential from lung carcinoma. Expression in RMC concurs with all studies except for the initial one by Tsuji [3], and expression in endothelial cells was previously identified by Naso et al. [4]. The novelty of the present study is in the analysis of a much larger series of breast and female genital carcinomas compared to previous reports. The current study also analyzed tubo-ovarian carcinomas that were classified based on current guidelines, which does not appear to be the case in the previous studies. Our data compellingly show that while HEG1 is useful in the differential diagnosis between mesothelioma and breast carcinoma, it is of little use in differentiating the former cancer from tubo-ovarian carcinoma. Despite the fact that mesotheliomas generally stained more diffusely for HEG1, its expression in 80/151 (53%) tubo-ovarian carcinoma effusions and 99/139 (71%) surgical specimens, respectively, precludes its use in this differential diagnosis.

In conclusion, analysis of a large series of malignant effusions confirms that HEG1 is a sensitive mesothelioma marker which performs well in the differential diagnosis from metastatic breast and lung carcinoma. However, it is not a useful marker in the differentiation of mesothelioma from tubo-ovarian carcinoma. Whether HEG1 can be used as a vascular marker awaits further research, though this avenue seems promising based on the present study.

## Data Availability

Raw data may be shared by the corresponding author upon reasonable request.
